# The Psychology Undergraduate Research Conference: A Pathway to Publishing?

**DOI:** 10.3389/fpsyg.2019.00491

**Published:** 2019-03-11

**Authors:** Christopher Kent, Peter J. Allen, Sam Harding, Jessica L. Fielding

**Affiliations:** ^1^School of Psychological Science, University of Bristol, Bristol, United Kingdom; ^2^Bristol Speech and Language Therapy Research Unit, North Bristol NHS Trust, Bristol, United Kingdom

**Keywords:** conference, undergraduate, publishing, engagement, authorship

## Introduction

The benefits associated with regularly engaging undergraduate psychology students in authentic research are widely recognized (Miller et al., 2008) and reflected in the learning goals and graduate attributes/requirements specified by psychology course accreditors worldwide. For example, UK accreditation standards (British Psychological Society, 2017, p. 12) state that students should graduate from an undergraduate psychology course able to demonstrate a range of research skills, and that such skills are best developed via engagement in a diversity of empirical experiences across the duration of the course. In the UK, the last and largest of these experiences is the undergraduate dissertation or research thesis project (Brewer et al., 2012). Accreditation is similar in the US (American Psychological Association., 2016) where psychology majors will have designed and conducted multiple research studies prior to graduation (Perlman and McCann, 2005; Stoloff et al., 2015). Although less ubiquitous in the US than in the UK, capstone research projects are growing in popularity, particularly in liberal arts colleges (Schermer and Gray, 2012; Chew, 2015).

Substantial infrastructure exists to support the supervision and conduct of undergraduate research. Considerably fewer resources are invested in its dissemination. Thus, for most students, submission of the project report or dissertation, which is intended for consumption by just one or two assessors, is the final stage of the research process (Garde-Hansen and Calvert, 2007). As noted by Kneale et al. (2016, p. 160), “although involvement in research is recognized as offering transformational experiences for undergraduates… the dissemination phase is generally underplayed.” In this paper we discuss the role that undergraduate research conferences can play in the dissemination of undergraduate research generally, as well as the specific role they can play in stimulating student-staff collaboration on publications developed from undergraduate research projects.

## Undergraduate Research Conferences

Undergraduate research conferences can offer students a forum for dissemination of their research findings and opportunities to “complete the research cycle” (Spronken-Smith et al., 2013, p. 105) through to, in some instances, a peer-reviewed publication. They also provide a mechanism through which students can practice communicating complex ideas and research findings. This is a graduate attribute for undergraduate psychology courses on both sides of the Atlantic (American Psychological Association., 2016; British Psychological Society, 2017). The ability to communicate effectively is a skill valued by employers (Appleby, 2018), and the communication skills of psychology graduates can advantage them relative to majors from other disciplines when competing for graduate level positions in many different industries (Halonen and Dunn, 2018). Preparing a talk for a lay psychology audience, rather than for assessment or a limited lab-group, provides students with experience of communicating complex ideas concisely and at a more general level of abstraction.

In the US, there are many local, regional and national undergraduate research conferences held annually (Miller et al., 2008). Educators have documented various conference models since at least the 1970s (e.g., Carsrud, 1975), and the number of such events has “exploded” in more recent years (Kierniesky, 2005). Preparing for and participating in such conferences promotes the development of research and communication skills, a sense of professional identity, self-efficacy, independence and collegiality (Stuber-McEwen and Thielen-Belveal, 2008; Helm and Bailey, 2013). Presenting at a conference correlates with graduate school attendance (Stoloff et al., 2015), and may provide students with the motivation and confidence to work with supervisors to develop their research into publishable papers (Seymour et al., 2004).

Compared to the US, opportunities for students to present at undergraduate research conferences in the UK are limited, and the literature on such opportunities is sparse. In 2011, Dancey et al. described an event where second year students were assessed on posters presented within the Second Life virtual world. Although described as a “conference,” the event would not correspond with most tacit definitions of this term. All 27 student attendees were members of the same class, participation was required, and the social networking opportunities inherent to face-to-face conferences were largely absent. Nevertheless, most students described the experience in positive terms. A non-virtual conference described by Lund (2013) aimed to “mimic the format and atmosphere of a professional conference” (p. 186). The conference could not achieve these aims because all delegates were from a single course, attendance was mandatory, and presentations were assessed. Nonetheless, most students valued it as both a learning experience and social event (Lund, 2013). Student delegates at the British Conference on Undergraduate Research (BCUR) shared similar sentiments (Kneale et al., 2016). Unlike the events described by Dancey et al. (2011) and Lund (2013), BCUR attendance is voluntary and open to students from across the UK. However, it is multidisciplinary, and only a small minority of 200+ presentations and posters at the 2018 event were delivered by psychology students (University of Sheffield, 2018). The development of conference presentations into publishable papers was not explored by Dancey et al. (2011), Lund (2013), or Kneale et al. (2016).

## The South West Undergraduate Psychology Conference

Our experiences of undergraduate psychology conferences are based on the South West Undergraduate Psychology Conference (SWUC). The SWUC, running for over 20 years, is a collaboration between six universities and the British Psychological Society (BPS). Hosting duties rotate and the conference attracts approximately 150 students and academics annually. The one-day event includes multiple oral streams and a lunch-time poster session, concluding with a keynote presentation by an established academic. A morning coffee-break and post-conference reception are scheduled to encourage networking between the students across institutions (see e.g., http://www.bris.ac.uk/psychology/news/2017/110.html). The event receives very positive feedback from both students and academics. Part of its success is attributed to bringing together students from different universities with diverse psychological approaches, to share their research experiences in a setting resembling a professional conference. For many students attendance is motivated by the opportunity to receive feedback about methodology and interpretation of their results which could improve their final thesis. The majority of the universities recognize attendance at the conference as extra-curricular professional development (http://www.bristol.ac.uk/careers/employable/plus-award/). The conference showcases some of the very best undergraduate research from the South West Universities, with all abstracts being published by the BPS (https://www.bps.org.uk/publications/south-west-review).

In recent years, the research presented at the conference by at least two undergraduate students has been published (Kent et al., 2014; Blackwell et al., 2018). Despite the authors' sense that both papers would have been published regardless of presentation at SWUC, what did the students gain from presenting, and could we further increase engagement in the publication process? Typically, the dissertation is only read by one or two examiners—one of which is the supervisor. The conference therefore offered the only other opportunity for communicating their research to a larger audience (given that this would be too early to consider publication). The conference thus helped to complete the research cycle for the students. As well as the inherent transferable skills involved in giving an oral presentation, informal discussion with previous presenters indicates that the conference reflects what they believe to be real academic conferences in structure and professional environment (including a conference booklet, refreshments, and dedicated conference staff), and thus offers insights into everything a professional conference involves. Although courses often require students to give assessed oral presentations, attendance and presentation at SWUC serves very different purposes, with different expectations and demands. Engagement with SWUC might signal that the student is motivated to pursue further academic study and, potentially, a career as an academic. This, in turn, facilitates a conversation with dissertation supervisors about engagement with the publication process. For those students not wishing to pursue a career in academia, the conference nonetheless offers an excellent opportunity to demonstrate to potential employers their transferrable skill of communicating complex ideas concisely to new audiences.

A barrier preventing SWUC from immediately leading to engagement in the publication process is the delay between data collection (and presentation at SWUC) and manuscript preparation. After students complete their dissertation there is typically an extended period before the supervisor (usually) instigates the publication process. Students will have moved on from undergraduate studies, which may limit the extent to which they are available and able to engage. We note that neither student is first author on the single experiment papers published subsequent to SWUC attendance (Kent et al., 2014; Blackwell et al., 2018). Although students do not have a full appreciation of their work until it is submitted in written form, a supervisor can gain perspective from the presentation at SWUC to help judge suitability for publication.

## Strategies to Strengthen the Pathway from Presentation to Publication

Academics typically attend conferences for a variety of reasons, including a desire for early feedback on research and networking opportunities (Sousa and Clark, 2017). Having not had a conference experience before, undergraduate students are not able to maximize the networking opportunities. One suggestion is that the undergraduate conference should not be an end point (or completion of the cycle) but rather a focal point for pre- and post-discussion of student work outside of the conference setting. Furthermore, academics will take their work to specialist conferences for expert feedback which can directly influence and help shape the manuscript. SWUC, however, spans the full range of research topics in both qualitative and quantitative methods. While feedback from a general audience may be difficult to directly apply to their work, at this stage in their development as researchers, this general feedback can provide students with alternative perspectives, allowing critical reflection on their work.

Considering the experience and insights provided by organizing and evaluating SWUC, building on the established good practice, we must consider what more undergraduate conferences could do in order to maximize the engagement of undergraduates in the publication process. One clear direction for future undergraduate conferences is to ensure pre- and post-event community building opportunities. This can be achieved through integration of online forums designed around the conference sessions. Using online forums would not only encourage students to independently seek out their own peer support networks but also enable inter-university collaboration, offer opportunities for peer-to-peer formative feedback and insight into alternative research perspectives that might directly impact on their project report. Forums can be run with a light touch affording minimal administration and could be set up in such a way that there are specific threads to stimulate discussion and consideration of engagement with publishable research. This creates a framework around which students can independently organize and manage their research, including dissemination and thinking about long-term publication plans. A further strategy to maximize the benefits of engaging with conferences would be to incorporate such experiences into the culture of studying for a BPS accredited undergraduate psychology course and the dissertation supervision process. If conferences were more fully incorporated into undergraduate studies as standard practice such events could be extended over a greater amount of time. This would allow for non-presenting activities such as informal networking events and conference dinners, both of which foster opportunity to collaborate and discuss research akin to professional conferences. Principally, additional time would allow for workshops to be held focusing on future careers, development of quality research and the publication process. As a final consideration, co-designing such experiences with students can offer great insight into avenues for further improvement on best practice, and allows students to develop other transferrable skills. It would be advisable when running future undergraduate research conferences to give students time to reflect on the conference process and the benefits of engaging with publishable research.

## Conclusion

Running SWUC demonstrates the worth of offering students an opportunity to attend inter-university psychology-focused conferences. Further improvements of this practice, as discussed, could enhance student engagement in publishable research. Adoption of this approach by other universities as a matter of standard practice could allow students and staff to capitalize on such an immersive experience increasing opportunity for more high-quality research and undergraduate engagement in the publication process.

## Author Contributions

All authors listed have made a substantial, direct and intellectual contribution to the work, and approved it for publication.

### Conflict of Interest Statement

CK and SH have previously been Chair and ordinary member, respectively, for the BPS South West Branch which helps organize the conference described in the article. The remaining authors declare that the research was conducted in the absence of any commercial or financial relationships that could be construed as a potential conflict of interest.
